# Speech therapy for exercise-induced laryngeal obstruction

**DOI:** 10.1007/s00405-024-09190-y

**Published:** 2025-01-21

**Authors:** Kristine Vreim, Tom Karlsen, Petter Helø Carlsen, Valgjerd Helene Nistad, Ola Drange Røksund, Thomas Halvorsen, Maria Vollsæter, John-Helge Heimdal, Hege Clemm, Mette Engan

**Affiliations:** 1 Department of Pediatric and Adolescent Medicine, Haukeland University Hospital, Bergen, Norway; 2 Faculty of Health and Social Sciences, Western Norway University of Applied Sciences, Bergen, Norway; 3 Department of Clinical Science, Faculty of Medicine, University of Bergen, Bergen, Norway; 4 Department of Sports Medicine, Norwegian School of Sport Sciences, Oslo, Norway; 5 Department of Otolaryngology and Head and Neck Surgery, Haukeland University Hospital, Bergen, Norway; 6 Department of Surgery, Haukeland University Hospital, Bergen, Norway; 7 Department of Clinical Medicine, University of Bergen, Bergen, Norway

**Keywords:** EILO, Laryngeal obstruction, Exercise, Dyspnea, Vocal Cord Dysfunction, Speech therapy

## Abstract

**Introduction:**

Exercise- Induced Laryngeal Obstruction (EILO) can lead to disabling exercise related dyspnea and hamper participation in physical activity. In this study, we aimed to investigate the effects of a standardized speech therapy protocol as treatment for EILO.

**Methods:**

Patients diagnosed with EILO at our institution were invited to participate. We compared laryngeal findings obtained during a continuous laryngoscopy exercise (CLE) test and questionnaire based self-reported breathing symptoms, before vs. after the treatment intervention. The laryngeal obstruction was characterized using a standardized CLE scoring system (0–12 points).

**Results:**

A total of 28 patients were evaluated. Following speech therapy, the mean reduction in the CLE score was 1.5 (95% confidence interval: 1.1–2.0) points, with the improvement primarily associated with decreased glottic-level obstruction. Twenty-four (86%) patients reported reduced symptoms during exercise. A moderate correlation was observed between changes in CLE scores and subjective symptom improvements.

**Conclusion:**

This study suggests that a standardized speech therapy protocol reduces observed laryngeal obstruction during the CLE test, with the most notable improvement occurring at the glottic level, alongside a parallel reduction in self-reported symptoms of EILO.

## Introduction

Exercise-Induced Laryngeal Obstruction (EILO) has a prevalence of 5.7–7.5% in the general adolescent population and is an important cause of exertional breathing problems, hampering participation in physical activities [1, 2]. Exercise induced dyspnea is the most common presenting symptom, accompanied by prolonged and most often noisy breathing, particularly in the inspiratory phase [3, 4]. Historically, EILO has been referred to by various terms, the most common being vocal cord dysfunction (VCD) [5]. By consensus, breathing problems caused by laryngeal narrowing in an otherwise normal larynx are now labelled inducible laryngeal obstruction (ILO), and EILO when exercise is the inducer [6]. In EILO, both supraglottic structures and the vocal folds (glottic level) may contribute to the narrowing of the larynx, and this heterogenicity likely calls for different treatment strategies [4, 7]. However, most treatment options for EILO currently build on weak evidence [8].

Traditionally, speech therapy has been considered mainstay therapy for ILO, and it is in many countries also considered the first line therapy for EILO [9]. In addition to speech therapy, current conservative treatments for EILO include simple breathing advice, biofeedback, and inspiratory muscle training [3, 10]. Speech therapy for EILO typically involves educating the patient about the nature of the condition, establishing lower thoracic breathing patterns, teaching rescue techniques to manage respiratory symptoms, and practicing the application of these techniques [9, 11–13].

Several studies have addressed the effect of speech therapy on EILO symptoms [9, 13–16]; however, the therapy methods used are often difficult to reproduce due to insufficient description, and outcome measures are typically based on self-reports using questionnaires [11, 17]. No studies have previously assessed its effect on visually determined laryngeal obstruction using the continuous laryngoscopy exercise (CLE) test.

At the outpatient clinic for EILO at Haukeland University Hospital, Bergen, Norway, speech therapy has been standardized and implemented as a treatment option for EILO [18]. This study aimed to evaluate the effect of this protocol by assessing both visually determined laryngeal obstructions and self-reported symptoms before versus after treatment.

## Methods

### Patients and study design

Since 2013, nearly all patients diagnosed with EILO at our institution have, by consent, been consecutively enrolled in the Bergen EILO-register at Haukeland University Hospital. The register includes demographic and medical data, breathing symptoms reported through a self-administered questionnaire, and visually assessed laryngeal observations obtained through continuous flexible laryngoscopy performed during treadmill exercise. This retrospective single-center observational study included all patients diagnosed with EILO who received speech therapy and successfully underwent CLE testing both before and after treatment between 2017 and 2021. Patients were excluded if they had undergone other treatment for EILO before the speech therapy. The Medical Research Ethics Committee of Western Norway approved the study (REK 2016/1898)**.** Informed written consent was obtained from all patients.

### CLE-test and spirometry

For assessment of lung function, a Vmax 22 © spirometer (SensorMedics, Yorba Linda, CA, USA) was used to perform spirometry according to guidelines [19]. A CLE test set-up that integrates a maximum cardiopulmonary exercise test and continuous video-recorded laryngoscopy, including sound recording and video recording of the upper part of the body, were applied for all patients. After applying local anesthesia (Xylocaine), a transnasal, flexible, fiberoptic laryngoscope with a diameter of 3.5 mm was introduced. The laryngoscope was secured using a custom designed helmet, and positioned to allow visualization of the laryngeal entrance, including the vocal folds and the supraglottic structures [7]. Continuous video-recorded laryngoscopy was performed throughout a maximum cardiopulmonary exercise test on a treadmill (Woodway ELG 55, Weil am Rhein, Germany) using a computerized modified Bruce ramp-protocol aiming to reach maximum exercise capacity after 6–14 min by increasing speed and elevation regularly [20]. Ergo-spirometry data were collected in conjunction with the CLE-test using a Jaeger Oxycon Pro Cardiopulmonary Exercise testing system (Viasys Health Care, Yorba Linda, CA, USA). The test was considered successful if the patient continued exercising until exhaustion or stopped because of intolerable respiratory distress.

### Evaluation of laryngeal obstruction

**Fig. 1:**
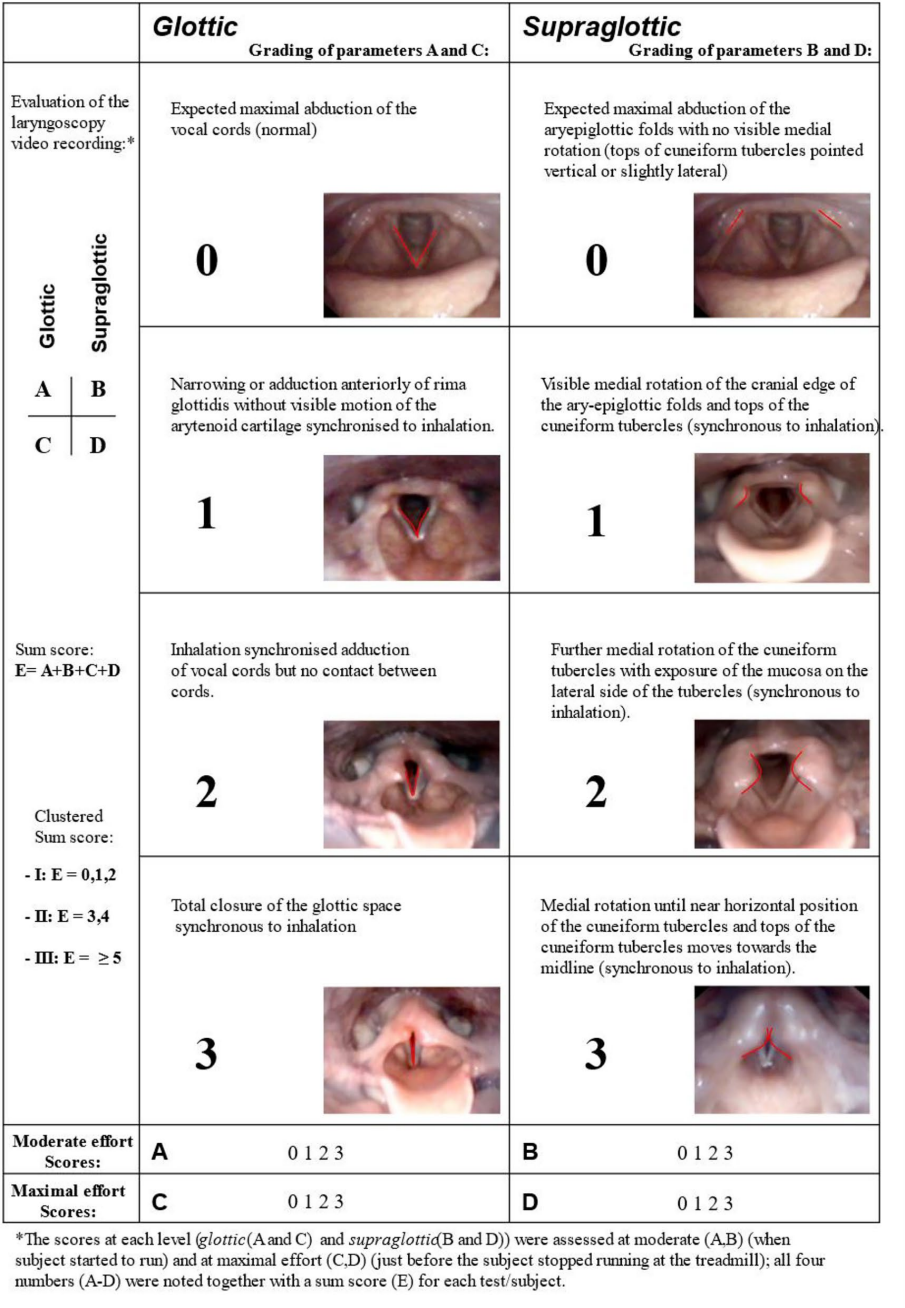
Grading system of laryngeal obstruction according to Maat et al., reproduced with permission

Two highly experienced raters (HC and ODR) scored all CLE tests according to a widely recognized and commonly used CLE scoring system [4]. The CLE scoring system visually grade the obstruction of the larynx, with scores from 0 (complete patency) to 3 (almost complete closure) at the glottic and supraglottic level at moderate and at maximum exercise intensity. The total CLE sum-score (E) was defined as the sum of all four sub-scores (A-D). Figure 1 provides a visual representation of the scoring system. The raters evaluated recordings from all patients retrospectively, blinded to whether the video had been obtained before or after the treatment period. Disagreements regarding scores were solved by consensus. A score at either glottic or supraglottic level ≥ 2 is commonly considered as a significant laryngeal obstruction and was interpreted as EILO [21].

### Treatment options for EILO at our institution during the inclusion period

First line therapy was physician-guided structured breathing advice while patients were observing their laryngeal responses on the monitor (biofeedback). This was received by all patients. Second line treatment options were speech therapy or physician-guided inspiratory muscle training (IMT). Supraglottoplasty was reserved for selected individuals with severe supraglottic EILO that typically experienced limited improvement with conservative treatment. Participants in this study were recruited from those who received speech therapy.

### The speech therapy protocol

The speech therapy protocol is based on general principals used in the treatment of voice problems, and was developed by one of the co-authors (TK, speech therapist) [18]. In the context of EILO, the goal is to achieve and maintain controlled and effective laryngeal abduction during exercise. The protocol focuses on reprogramming the involved muscles, desensitizing patients to triggers, and implementing diaphragmatic breathing to support laryngeal patency [22]. During the first session, the participants are presented with information about the anatomy of the larynx and the pathophysiology of EILO. The patients undergo five sessions with a trained speech therapist, with each session introducing a new set of breathing exercises. The sessions were conducted in two modes, either scheduled approximately one week apart over five weeks or delivered intensively over three consecutive days.

#### First session, Set 1

The objectives of this set are for the patient to achieve diaphragmatic breathing, reduce muscle tension, and generate positive airway pressure to widen the larynx.

Exercise 1: “Tube-technique”, blowing through a wide tube into a water-filled bottle, to reduce tensions in the larynx and generate positive airway pressure to widen the larynx during expiration.

Exercise 2: “F-out, yawn in", requires slowing down the airflow during expiration with the mouth partially closed, as in forming an “F-sound,” and inspiration with a yawn-like mouth figuration.

#### Second session, Set 2

The objectives of this set are to maintain diaphragmatic breathing and reduce tensions with various inspiration variations, and to create resistance and trigger laryngeal abduction reflexes.

Exercise 1: “Tube-technique”.

Exercise 2: “F-out, yawn in”.

Exercise 3: “F-out, sniffing in”, similar expiration to the second exercise, but the air is sniffed in through the nose during inspiration.

Exercise 4: “F-out, lateral mouth extension in”, similar expiration, but inspiration with closed teeth and expanded corners of the mouth.

Exercise 5: “Dew-out, dew-in”, instructs the patient to expire as if fogging an imaginary window and inspire without changing the articulatory position.

#### Third session, Set 3

Introducing stridor, the sound of adducting vocal folds during inspiration. The objectives of this set are desensitization and to make the patient aware of the differences between adduction and abduction in the larynx.

Exercise 1: “Tube-technique”.

Exercise 2: “F-out, stridor in; f-out, yawn in”’.

Exercise 3: “F-out, stridor in; f-out, sniffing in”.

Exercise 4: “F-out, stridor in; f-out, lateral mouth extension in”.

Exercise 5: “F-out, stridor in; dew-out, dew-in”.

#### Fourth session, Set 4

The objective of this set is to achieve a swift controlled switch from adduction to abduction, switching from stridor to one of the opening exercises.

Exercise 1: “Tube-technique”.

Exercise 2: “F-out, start inhaling stridor in, switch and inspire as sniffing”’.

Exercise 3: “F-out, start inhaling stridor in, switch and inspire with lateral mouth extension”.

Exercise 4: “F-out, start inhaling stridor in, switch and inspire as yawn”.

#### Fifth session, Set 5

The objective of this set is to maintain diaphragmatic breathing and laryngeal control while gradually increasing exercise intensity. The fifth set consists of three exercises in addition to gradual implementation of the techniques during physical activity in pyramid structured training sessions.

Exercise 1: “Tube-technique”.

Exercise 2: “F-out, yawn in”.

Exercise 3: “F-out, lateral mouth extension in”.

The sets are rehearsed during the periods between the treatment sessions and the patient must adequately practice and master it before a new set is introduced. Patients are encouraged to perform the exercise set they learned the previous session, preferably with 15 breathing cycles per exercise, repeating these exercises multiple times. A thorough description of the content and development of the speech therapy treatment approach has recently been published [18].

### Self-reported subjective symptoms

A questionnaire used in our EILO clinic for several years was administered to assess the impact of breathing symptoms on participants’ overall life and physical activity, with changes in scores before versus after treatment serving as the outcome.

### Statistical methods

This was a retrospective observational study with changes in CLE-scores before vs. after speech therapy as the primary outcome. The secondary outcomes were changes in self-reported symptom scores. Baseline characteristics were presented as means with standard deviations (SDs). The four CLE sub-scores obtained at moderate and maximum intensity at glottic and supraglottic level, were ranged from 0 to 3 and then added together to calculate the CLE sum-score. The mean changes in CLE scores and symptom scores were calculated using students paired t-test and reported with 95% confidence intervals (CIs). The CLE scores and symptom scores are ordinal and categorical variables, respectively; however, due to the low number of categories, the data were nevertheless reported as mean values and mean differences with 95% CIs, as this is considered to provide more information than medians and interquartile ranges in such situations [10, 23]. Correlation between change in CLE sum-score and change in self-reported subjective improvement after speech therapy (improvement: 1 = no improvement, 2 = some improvement, 3 = much improvement, 4 = complete recovery) was calculated using Pearson correlation coefficient. The Chi-Square test was used to assess changes in proportions. Statistical analyses were conducted with IBM SPSS Statistics version 29.

## Results

During 2017–2019, 40 patients referred for EILO assessment performed CLE-testing and thereafter received speech therapy before a second CLE-test. For 10 patients, video recordings were either missing or of too poor quality. Two patients had received alternative treatment, and one of these lacked ergo-spirometry data. These 12 patients were excluded, which left 28 patients (1 male) eligible for inclusion. Baseline characteristics of the patients are outlined in Table 1. The patients were between 16–39 years and attended different types of sports like football, handball, and cross-country skiing. The mean (SD) time between first and second CLE-test was 17.8 (8.1) months.

**Table 1:** Baseline characteristics of 28 included patients from the EILO register at Haukeland University Hospital, Bergen, Norway who were treated with speech therapy for EILO during 2017–2021

Variables	N = 28
	Pre speech therapy
Female, *n (%)*	27 (96.4)
Age in years, *mean (min–max)*	16.5 (12–39)
Height in cm, *mean (SD)*	167.0 (8.0)
Weight in kg, *mean (SD)*	59.6 (8.4)
Time between CLE-tests, *mean, (SD)*	17.8 (8.1)
FVC, % predicted, *mean (SD)*	102.6 (11.6)
FEV_1_, % predicted, *mean (SD)*	106.9 (12.9)

Abbreviations: *FVC* forced vital capacity, *FEV*_*1*_ forced expiratory volume in first second time, *SD* standard deviation

### Laryngeal findings during exercise, CLE-scores

None of the subjects had any structural laryngeal abnormalities at rest. The distribution of CLE sub-scores is presented in Table 2. This includes sub-scores at the glottic and supraglottic level at moderate and maximum exercise intensity, and the CLE sum-score. Before treatment, all patients had a CLE sum-score of ≥ 3 and at least one sub-score of ≥ 2. At maximum exercise, the supraglottic score exceeded the glottic score in 25/28 patients, 6 had a glottic obstruction score of one or less, while 2 had a supraglottic score of one or less.

**Table 2:** Number of patients according to change in CLE-sumscore (E) and CLE-score at maximum exercise intensity, glottic (C) + supraglottic (D) level after speech therapy

Change in CLE- score	CLE-sumscore		Change in CLE- score	CLE-score at maximum exercise intensity	
	n	%		n	%
**0**	4	14.3	**0**	7	25.0
**1**	12	42.9	**1**	12	42.9
**2**	8	28.6	**2**	7	25.0
**3**	2	7.1	**3**	2	7.1
**4**	1	3.6	**4**	0	0
**5**	1	3.6	**5**	0	0
**Sum**	**28**	**100**		**28**	**100**

Abbreviations: CLE-sumscore, E; CLE-score at maximum exercise intensity, C + D (see Fig. 1)

At the second CLE-test, conducted after speech therapy, none of the patients showed an increase in their CLE sum-score, 24/28 (86%) patients had a reduction of 1 point or more, and 12/28 (43%) had a reduction of 2 points or more (Table 2).

**Fig. 2:**
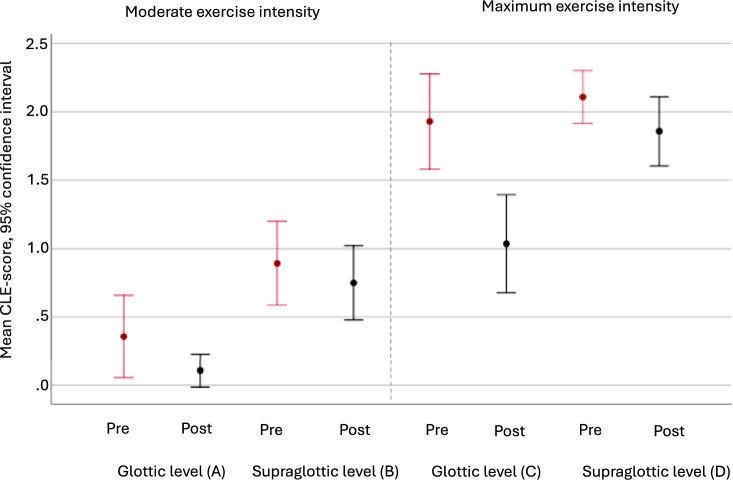
Mean CLE subscores with 95% confidence intervals at moderate and at maximum exercise intensity pre and post speech therapy in the 28 included patients from the EILO register at Haukeland University Hospital, Bergen, Norway

The mean changes in CLE scores are reported in Fig. 2 and Table 3. The mean reduction in CLE sum-score was 1.54 (95% CI: 1.08, 1.99) points (p < 0.001). Regarding the sub-scores, the largest improvement was observed at the glottic score at maximum exercise, with a mean change of 0.89 (95% CI: 0.59, 1.20) points, (p < 0.001).

**Table 3:** Continuous laryngoscopy exercise (CLE) scores from the 28 included patients who were treated with speech therapy for EILO during 2017–2019 in the EILO register at Haukeland University Hospital, Bergen, Norway

CLE score	Pre treatment	Post treatment	Change pre-post^a^		p value
	Mean (SD)	Mean (SD)	Mean	95% CI	
Moderate exercise intensity					
A: Glottic level (0–3)	0.4 (0.78)	0.1 (0.32)	0.25	(0.02, 0.48)	0.03
B: Supraglottic level (0–3)	0.9 (0.79)	0.8 (0.70)	0.14	(0.01, 0.28)	0.04
Maximum exercise intensity					
C: Glottic level (0–3)	1.9 (0.90)	1.0 (0.92)	0.89	(0.59, 1.20)	< 0.001
D: Supraglottic level (0–3)	2.1 (0.50)	1.9 (0.65)	0.25	(0.08, 0.42)	0.01
**E: Sum score (0–12)**^**b**^	**5.3 (1.80)**	**3.8 (1.62)**	**1.54**	**(1.08, 1.99)**	** < 0.001**

Abbreviations: *CI* confidence interval, *SD* standard deviation

^a^Estimates from Student’s paired t-test

^b^See Fig. 1 for explanation of CLE sum-score

### Subjective symptom outcomes

Table 4 presents the mean changes in symptom scores. Following speech therapy, we observed a significant improvement in the impact of breathing problems on overall quality of life and physical activity. In total, 4/28 (14%) patients reported no improvement, 20/28 (86%) reported some or much improvement, and 4/24 (14%) reported complete resolution. The rate of patients reporting that their breathing problems during exercise bothered them a lot or was of a disabling character, changed from 18/27 (67%) to 11/27 (41%) (p = 0.06) from before vs. after treatment. The rate of patients reporting that their breathing problem overall in their life bothered them a lot or was of a disabling character, changed from 9/27 (33%) to 6/27 (22%) (p = 0.36). The rate of patients reporting that their EILO symptoms prevented them from exercise always or almost always, changed from 8/27 (30%) to 3/27 (11%) (p = 0.09).

**Table 4:** Self-reported breathing symptoms in 27 of the 28 included patients who were treated with speech therapy in the EILO register at Haukeland University Hospital, Bergen, Norway

Question	Pre-treatment	Post-treatment	Difference pre-post treatment^a^	p value
	Mean (SD)	Mean (SD)	Mean (95% CI)	
*Q1. “Overall, in your life, how much are you bothered by your breathing difficulties?” (1–5)*	3.0 (1.0)	2.3 (1.3)	0.7 (0.06, 1.28)	0.03
*Q2. “During physical activity, how much are you bothered by your breathing difficulties?” (1–5)*	3.8 (0.9)	2.9 (1.3)	0.9 (0.21, 1.50)	0.01
Q3. “*My symptoms prevent me from exercising”. (1–5)*	2.7 (1.3)	1.9 (1.1)	0.9 (0.21, 1.57)	0.01
*Q4.”* How have your symptoms changed after the treatment period?				
No improvement, n (%)		4 (14)		
Some improvement, n (%)		9 (32)		
Much improvement, n (%)		11 (39)		
Complete improvement, n (%)		4 (14)		

Abbreviations: *EILO* exercise induced laryngeal obstruction, *NRS* numeric rating scale, *SD* standard deviation, *CI* confidence interval

^a^Estimates from Student’s paired t-test

### Correlations between laryngeal findings and subjective symptom outcomes

**Fig. 3:**
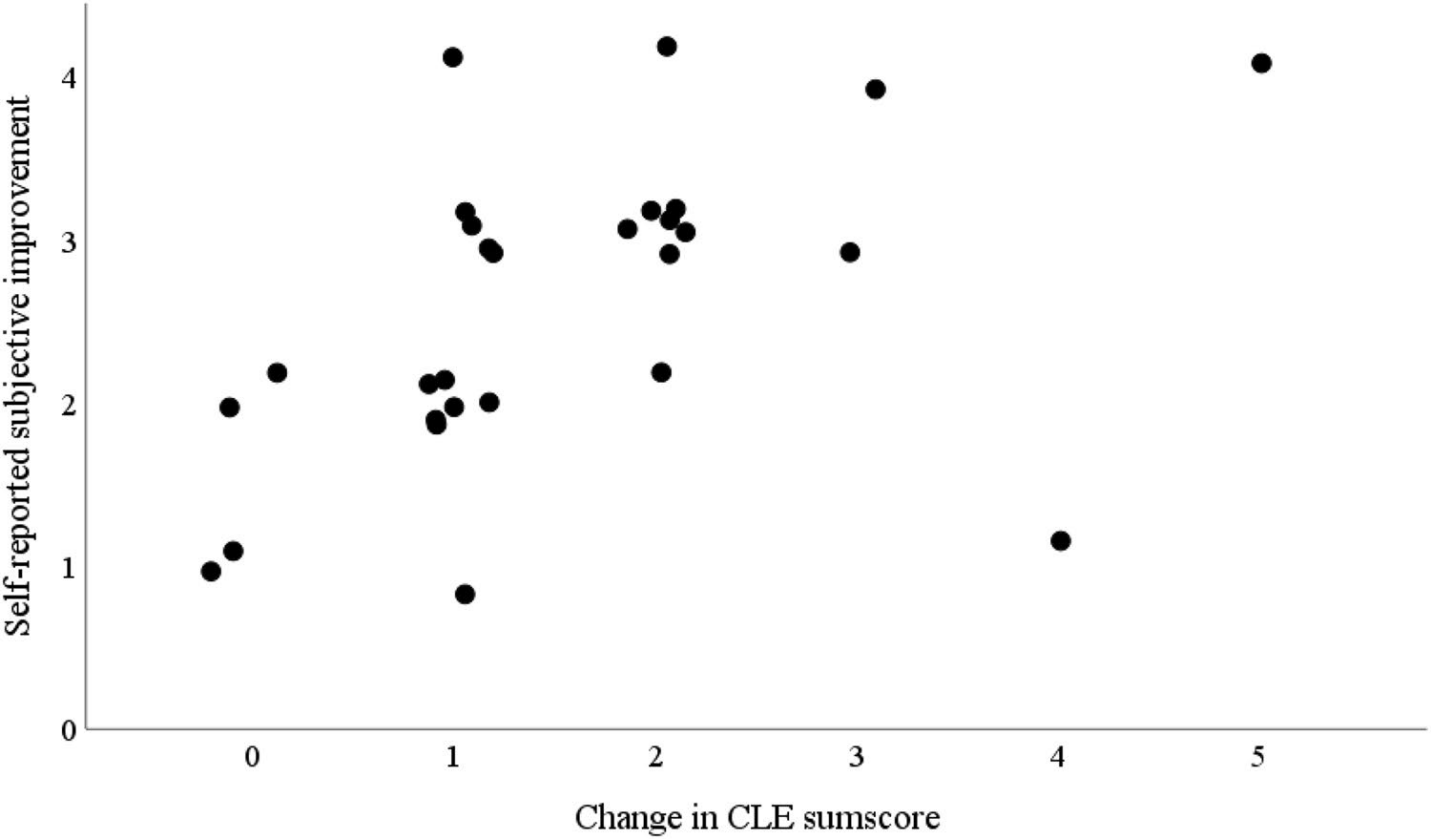
Correlations between Self-reported subjective improvement and Changes in CLE sumscore in the 28 patients after speech therapy. Self-reported subjective improvement: 1 = no improvement, 2 = some improvement, 3 = much improvement, 4 = complete recovery. Change in CLE sumscore = CLE sumscore before treatment – CLE sumscore after treatment. Pearson correlation coefficient: 0.445, p = 0.18

Correlations between laryngeal findings (CLE-scores) and subjective symptom scores are illustrated in the scatterplot in Fig. 3. The Pearson correlation coefficient was 0.45 (p = 0.18), indicating a moderate correlation between the two measures [24].

## Discussion

This is the first study to evaluate CLE scores as well as self-reported symptoms before versus after standardized speech therapy in EILO patients. Most patients showed improvements in both measures, with the greatest laryngeal improvement observed at the glottic level, highlighting the physiological impact of speech therapy. The findings suggest that the treatment protocol used may be effective for treating EILO, although further controlled studies are needed to confirm these results.

Our results are in line with previous studies reporting recovery or complete resolution of EILO symptoms after speech therapy [9, 13, 15, 25] Chiang et al. found that 72% of the 18–81-year-old subjects experienced improvement or complete resolution of their EILO symptoms after laryngeal control therapy [15]. In studies examining adolescents, speech therapy has been reported to control symptoms of EILO six months post therapy in 95% of subjects [16]. Additionally, the proportion of participants reporting that they often or always have to stop physical activity due to breathing problems have been reported to decrease from 56 to 11% [13]. Direct comparison between studies is challenging due to variations in patient populations and speech therapy strategies. However, comparing different speech therapy protocols and identifying factors associated with varying levels of effectiveness would be valuable for developing personalized treatment approaches.

Previous studies have applied various speech therapy approaches to treat EILO. Our speech therapy protocol consisted of five sessions. Previous reports have suggested that two-six sessions would be a reasonable number of sessions to improve EILO symptoms, with the highest estimates for patients in whom EILO had led to reduced physical activity [9, 26]. Diaphragmatic breathing is foundational for EILO symptom management [9]. Other described techniques in line with the assessed protocol include sniff inspiration, a prolonged expiration cycle, and tension reduction [9, 15]. One of the main differences from our study, is the standardization of the techniques. Shaffer et al. suggests that effective treatment should be tailored to the individual patient based on clinical discussions and the symptom severity, which is complicated by a standardized approach. Considering the heterogeneity of EILO patients, we could assume that better results may be achieved by making such adjustments. However, a standardized approach is favorable in research because we can know that similar treatment is received by all patients. Additionally, standardized treatment enhances accessibility and reproducibility.

This study demonstrates that speech therapy could help most patients with EILO maintain an open larynx, even at peak exercise. Currently, other treatment options for EILO includes inspiratory muscle training (IMT) and supraglottoplasty. Effects of IMT and supraglottoplasty have been promising, with improvements reported in approximately 80% of subjects [10, 27]. At present, we have limited knowledge about which treatment is best suited for each individual patient [8]. The results of this study suggest that speech therapy is more effective at the glottic compared to the supraglottic level. This study recruited all patients with EILO, irrespective of subtypes, and it is reasonable to assume even more favorable outcomes if predominantly glottic EILO had been included.

A strength of this study lies in the integration of both subjective symptoms and visually observed laryngeal obstruction in the evaluation of outcomes. By combining self-reported symptoms with the CLE-scores, we were able to assess the treatment from different perspectives. Although the CLE-test is considered the gold standard in assessing EILO, the scoring system has its shortcomings [21]. Despite being conducted by experienced raters, the scores are inherently based on partially subjective evaluations of observed laryngeal narrowing, and previous studies have yielded mixed results regarding the validity of the CLE scoring system [4, 21, 28]. Nonetheless, the CLE-test remains essential for diagnosing EILO and determining its topography and severity [29].

Correlation analyses revealed a moderate correlation between the CLE-test results and the subjective symptom score. This may argue against the necessity of a second CLE test in patients reporting significant improvement. However, until more advanced diagnostic tools are validated, we will still recommend a comprehensive evaluation that includes both subjective assessments and laryngeal examinations.

A limitation of this study is its single-center, retrospective, non-controlled design. The improvements could be attributed simply to the reassurance patients felt from receiving a diagnosis or to the physician-guided structured breathing advice with biofeedback provided to all patients during the diagnostic session, with an unknown contribution from the speech therapy. Another limitation was the lack of information on patients’ adherence to the protocol and the prescribed home exercise regimen. The treatment protocol was quite comprehensive, and it is possible that some patients did not complete all exercise repetitions as instructed. Another confounding factor could be that the time between the first and second CLE tests varied among patients.

Finally, research indicates no gender difference in the prevalence of EILO before puberty, but a higher prevalence in females after puberty [3]. This study predominantly included young females, with only one male participant, limiting the generalizability of the findings.

Properly controlled studies are needed to further investigate the effects of speech therapy as a treatment for EILO. The results of this study can serve as a foundation for future randomized trials exploring treatment options. The ongoing HelpILO study will provide valuable insights in that respect [30].

## Conclusion

This study indicates that a standardized speech therapy protocol improves self-reported symptoms of EILO and reduces observed laryngeal obstruction during a CLE test, with the most notable improvement occurring at the glottic level. These outcomes were correlated, suggesting a link between symptom relief and objective measures of laryngeal function. Future controlled interventional studies are needed to further validate these findings.

## Data Availability

Following the approvals granted for this study by The Regional Committee on Medical Research Ethics and The Norwegian Data Inspectorate, the data files are stored securely and in accordance with the Norwegian Law of Privacy Protection. The data file cannot be made publicly available as this might compromise the respondents’ privacy. A subset of the data file with anonymized data can be made available to interested researchers upon reasonable request to Hege Clemm (hsyh@helse-bergen.no), providing Norwegian privacy legislation and the General Data Protection Regulation (GDPR). GDPR is respected and the permission is granted by The Norwegian Data Inspectorate and the data protection officer at Haukeland University Hospital.
